# Vaccine risk assessment in children with a referred reaction to a previous vaccine dose: 2009–2011 retrospective report at the Bambino Gesu’ children hospital, Rome, Italy

**DOI:** 10.1186/1824-7288-40-31

**Published:** 2014-03-31

**Authors:** Luciana Nicolosi, Annachiara Vittucci, Rossella Mancini, Elena Bozzola, Alberto Cagigi, Annalisa Grandin, Alberto Villani

**Affiliations:** 1 Pediatric and Infectious Diseases Unit, Pediatric Medicine Department, Bambino Gesù Children’s Hospital, IRCCS, Rome, Italy; 2 Faculty of Medicine and Surgery, La Sapienza University, Rome, Italy; 3 Immunology and Infectious Diseases Unit; University Hospital Pediatric Department, Bambino Gesù Children’s Hospital, IRCCS, Rome, Italy

**Keywords:** Vaccination, Children, Side effects

## Abstract

**Background:**

During the last century, mass vaccination programs have achieved considerable success across the world in immunizing against several serious infectious diseases. However, vaccinations are threatened by their own success after results have been obtained: the more the incidence of potentially devastating diseases decreases, thanks to the success of vaccination programs, the more public attention shifts towards real or alleged “side effects” of vaccines.

**Methods:**

We analyze the experience of 153 children with “reaction to a previous vaccine dose” continuing the vaccination protocol in the safe environment of the Center for risk vaccination at the Bambino Gesù Children’s Hospital IRCCS in Rome, from 2009 to 2011.

**Results:**

To assess the suitability for vaccination, a specialized pre-vaccination advice and a skin prick test (SPT) was undergone, according to Wood’s guideline; 151 children were SPT negative and full vaccine was administered. Of the 153 children examined just 13 had symptoms suggestive of IgE-mediated reaction-type reactions with angioedema manifestations. Among them, 2 had positive STP, which required alternative measures of administration of the vaccine. No cases of post vaccination reaction was reported and no vaccination program was stopped due to a severe reaction.

**Conclusions:**

Inadequate levels of immunization against infectious diseases remain a significant problem for public health. However, the reasons for incomplete vaccination and non-adoption of vaccination services are manifold. To maintain public confidence in vaccines, advanced immunization programs must include activities for monitoring the safety of the vaccine at the individual level and pursuing specialized counseling pre-and post-vaccination for those at risk. Our results underlined a gap between true and referred adverse reactions and are consistent with vaccine safety. Anyway, a continuous assessment of the risks and benefits of vaccination is required and the results must be disclosed in order to strengthen confidence in the existing and in the new immunization programs.

## Background

Since the days of Jenner and Pasteur, inducing an immune response to infectious diseases by the way of vaccination has become a widely applied intervention to keep people healthy. Globally, the population coverage of vaccination programs has expanded so that immunization has served to eradicate potential fatal diseases, such as smallpox. Moreover, vaccination has stickling reduced the morbidity and mortality due to childhood infectious diseases in developed countries and also protected infants too young to be vaccinated [1]. Nevertheless, parents may be still hesitant to get their children vaccinated due to a lack of knowledge of the seriousness of the disease, skepticism about the vaccination benefits, and increased fear of adverse events following immunization. In order to implement vaccination coverage, misinformation regarding vaccines must be addressed promptly. In particular, to improve vaccination, healthcare providers should take advantage of every healthcare visit as an opportunity to evaluate vaccination status and administer vaccines when needed [2]. Declining vaccination rates should facilitate the spread of illness and death from vaccine-preventable diseases and should increase costs for society, both in direct health care costs and indirectly in lost productivity [3].

Despite currently available vaccines are safe [4,5], mild adverse reactions are occasionally observed. Severe reactions such as anaphylaxis [6] may occur in rare cases (0,26 case per 1000000 doses).

These are generally due to the components contained in vaccines such as egg protein, gelatin, antibiotics and other potential additives, rather than to specific antigens.

The approach to the patient starts with an evaluation of the symptoms and of the timing of their appearance from vaccination. The immediate reactions are generally easier to identify and to attribute to the hypersensitivity to vaccine for both the timing and the characteristic symptoms of the allergic reaction. Moreover, being IgE-mediated, causative agent can be identified by skin prick tests (SPT) or in vitro by specific IgE assay. Skin tests and/or search for specific serum IgE are extremely useful for evaluating the patient's risk of relapse: this is because the reactions to the next dose of the same vaccine may be more immediate and severe than the first time and, eventually life threatening [7].

Delayed-type reactions are generally much more difficult to attribute to a vaccine [8]. Most of the signs and symptoms are nonspecific and may be caused by other factors, including intercurrent infections. Patients should therefore be carefully evaluated in order to exclude other possible causes before any conclusion. Since the delayed reactions are not IgE-mediated, skin tests or in vitro studies are not useful to identify the causative agent or the responsible vaccine component.

Other laboratory tests are not reliable in the diagnosis of non-IgE-mediated hypersensitivity reactions. The decisions regarding patients with suspected delayed-type reaction must be left to the clinical judgment in most situations. Although non-IgE-mediated delayed reactions may be important, they rarely put the patient in severe life-threatening. The occurrence of a delayed vaccine reaction does not predispose to an immediate reaction to the next dose.

Delayed reactions occur from hours, up to days after exposure. The longest possible interval between exposure and the onset of symptoms is not completely clear, although most immunologists agree that reactions may occur up to 2 or 3 weeks after exposure. Most delayed reactions are classified as type 3 hypersensitivity and due to the formation of immune complexes, although other less well-defined mechanisms including T cell-mediated processes (type hypersensitivity 4), may also play a role [9].

Therefore, according to World Allergy Organization classification [10] (WAO), immunological reactions to drugs (including vaccines) are distinguished according to the time of onset of symptoms in:

1. immediate reactions that may start from a few minutes to 1–2 hours after administration of the vaccine, and are IgE-mediated.

2. delayed reactions that may occur from several hours up to days after administration. These reactions can be caused by different mechanisms, but they are rarely IgE-mediated.

A combination of several signs and symptoms (up to 40) characterize the immediate reactions (IgE-mediated); the most common signs and symptoms are:

• Skin symptoms, including hot flashes, itching, urticaria, angioedema

• Respiratory symptoms, including nasal secretion and congestion, change in voice quality, sensation of throat closure or choking, stridor, coughing, wheezing and dyspnea

• Cardiovascular symptoms, including weakness, syncope, altered mental status, palpitations, hypotension.

• Gastrointestinal symptoms, including nausea, cramps or abdominal pain, diarrhea and dysphagia.

The most severe form of IgE-mediated reaction is anaphylaxis, defined as a systemic allergic reaction with rapid onset and worsening which can lead to the patient's death [11,12]. Anaphylactic reactions to vaccines are very rare (0,26 case per 1000000 doses) [6].

Delayed reactions may be of immunological or non-immunological type [13]. Fever and irritability, local reactions to vaccination, such as swelling and redness at the injection site, are common and self-limiting and should not preclude further doses of vaccine. Other delayed reactions include rare cases of erythema nodosum [14] and encephalopathy; some of these reactions may be contraindications to further doses of specific vaccines.

Vaccine administration may cause vasovagal reaction (fainting) in certain patients who are prone to this response. Vasovagal reactions are characterized by hypotension, pallor, sweating, weakness, nausea, vomiting, bradycardia, and if severe, loss of consciousness.

Vasovagal reactions are able to simulate anaphylaxis, because both can lead to hypotension and collapse [15]. However, fainting is usually preceded by pallor, while anaphylaxis often begins with hot flashes and may also include itching, hives and angioedema. In anafilaxis, there is more often tachycardia than bradycardia.

In patients who reported fainting in response to vaccination, it is cautious to administer vaccines in supine position.

No action is absolutely risk free. As for associated vaccination risks, we must distinguish between real and perceived risks. Differentiation between coincidence and causality is of utmost importance in this respect. To assert that a given event is caused by vaccination, it is especially necessary to rule out other causes that may come to a diagnosis of exclusion. The timing of symptoms appearance from vaccination is often determinant. Severe allergic vaccine reactions are very rare and difficult to predict.

To maintain public confidence to vaccines, advanced immunization programs must include activities for monitoring safety of vaccines at the individual level, as well as pursuing specialized pre-and post-vaccination counseling for subjects at risk [12,16].

## Methods

Since 2004, at the Bambino Gesù Children’s Hospital IRCCS in Rome, a Centre for risk Immunization is active as part of the General Pediatrics and Infectious Diseases Unit, in the Department of Pediatric Medicine.

This center welcomes the growing demand of the Local Health Agencies of Rome and Lazio and of the pediatricians and families, to vaccinate children in a “safe environment” facing a number of challenges and allergy risks; the incoming patients who access to the Centre may have food allergy, previous reactions to vaccine doses, chronic persistent asthma, personal history of anaphylaxis, family history of anaphylaxis, chronic pulmonary, cardiac, renal, neoplastic pathology who have difficult clinical management, congenital and acquired immunodeficiencies and solid organ transplant recipients receiving immunosuppressive therapy, candidates for solid organ transplant or bone marrow transplant, splenectomy, requiring personalized vaccination calendars. The conditions of admission to the Centre are both free demand and prescription by an health care provider.

A specialized pre-vaccination advice for all the children is carried out to assess their suitability for vaccination, following an anamnestic report; possible solutions to safely continue vaccinations of children are then proposed to the families, following vaccination programs tailored to the underlying disease and the reason for directing the children to the Centre. All this is fully consistent with the absolute, temporary and false contraindications and precautions listed for the individual case.

The data were extrapolated from the retrospective analysis of discharge records of Day Hospital for risk vaccination’s hospitalizations. The number of accesses and not the number of children has initially been reported, as the same child might have accessed the center several times to complete the schedule, for different reasons. Among these reasons, we summarized those sent for “reaction to a previous vaccine dose”.

Analyzing each case, it was observed that children were directed to the Center several times for the same reason and for subsequent different vaccines. The following tables will therefore refer to the number of children and no longer to the reason for access.

The reasons for access were recorded according to reports by parents as very few cases had a report of the Local Health Agencies, vaccinating physician or pediatrician or a possible emergency physician; therefore, based on the history reported by parents, groups were defined according to a more conventional terminology. No specific and validated questionnaire has been used to collect data.

Each child was subjected to the procedure provided for the purpose: skin prick test (SPT) with a full drop of vaccine in contemporary with histamine SPT as a positive control and saline as a negative control has been made; if the reading of the SPT is negative, we proceed to the full administration of the vaccine; if the SPT is positive (positive is considered a wheal of 3 mm > negative control) and a further dose of the vaccine is required, after discussion with the parents on the risks/benefits budget of vaccination, and after their consent, we proceed to the administration of the vaccine by the method of desensitization, with 3–4 escalating doses of the vaccine every 15–30 minutes. The child remains under observation for the next 3 hours; authorities monitor vital parameters prior to vaccination, 30 minutes after vaccination and after 3 hours [9,17].

## Results

938 accesses have been recorded from 2009 to 2011 in the Center for risk vaccination.

In Table 1 we summarized those sent for “reaction to a previous vaccine dose”, a total of 286 accesses (30,5%), represented by 153 children (53%), each child sending almost 2 times for the same reason and for subsequent different vaccines. Children were 79 males and 74 females.

**Table 1:** Accesses and children sent for “reaction to a previous vaccine dose”

**YEAR**	**n° access**	**n° children**
2009	66	38
2010	97	49
2011	123	66
TOT	286	153

The average was of 3 years (range 4 months - 16 years); the median age was of 15 months.

In Table 2 are listed the previous dose of vaccine reactions. In Table 3, we sampled such vaccines causing the reaction. For 4 children, it has not been possible to trace the vaccine responsible for the reaction as not noted in the card discharge.

**Table 2:** Previous dose of vaccine reactions

**Previous dose vaccine reaction**	**n° children**
Urticaria	28
Fever and/or hypotonia	20
Anaphylaxis or angioedema	16
Local or generalized edema	15
Convulsions or clonus	12
Fever	12
Fever and urticaria	9
Dyspnea	7
Hyporesponsiveness	7
Hypertonicity	5
Uncontrollable crying or irritability	5
Thrombocytopenia	3
Vomiting	3
Fever and vomiting	2
Petechiae at the injection site	2
Hypothermia	1
Local abscess	1
Headache, paresthesia and dyslalia	1
Cyanosis	1
Fever and local petechial	1
Lymphadenopathy	1
Local vasculitis after 8 days	1

**Table 3:** Vaccines causing the reaction

**Vaccine**	**n° reactions**
Hexavalent and pneumococcus	62
Hexavalent	50
Pneumococcus	9
Measles-mumps-rubella (MMR)	8
Unknown	4
Papillomavirus	4
Poliovirus	1
Diphtheria-tetanus- poliovirus (DTPa)	1
Flu	2
Meningococcus	2
Pentavalent + hepatitis B (HBV)	2
Poliovirus + DTPa	2
Combined pentavalent + HBV + pneumococcus	1
Tetanus	1
Diphtheria-tetanus	1
Hexavalent + MMR	1
Hexavalent + palivizumab	1
Meningococcus + MMR	1

In Table 4 we classified the reactions caused by administrations of hexavalent vaccine, its co-administration with pneumococcus conjugate vaccine, pneumococcal conjugate vaccine and measles mumps rubella vaccination (MMR) vaccine.

**Table 4:** Reactions caused by vaccines

**Type of reaction**	**Type of vaccine**			
	**Hexavalent + Pneumococcus**	**Hexavalent**	**Pneumococcus**	**MMR**
Urticaria	12	4	3	4
fever and/or hypotonia and/or hyporesponsiveness	11	13		1
Anaphylaxis/Angioedema	6	5	2	
Febrile convulsions/Clonus	5	5	1	1
Diffuse erythema	5			
Fever ≥ 39 °C	5	5		2
Lower limbs swelling	4	4		
Dyspnea	3	2		
Vomiting	3	2		
Hypertonic crisis	2			
Fever and urticaria	2	6		
Fever and/or irritability and/or uncontrollable crying	1	2	1	
Headache/Paresthesia		1	1	
Petechiae	1		1	
Thrombocytopenia	1	1		
Vasculitis 8 days later	1			
Total	62	50	9	8

20 children had an underlying disease, including thrombocytopenia (2), lue connatal (1), atopic dermatitis (1), chromosomopathy with gastroesophageal reflux disease (RGE) (1), broncodisplasia (1), RGE (2), renal hypoplasia with vesicoureteral reflux (VUR) (2), DiGeorge syndrom with RGE (1), myoclonic infant epilepsy (1), tuberous sclerosis with West syndrom (1), spherocytosis with splenectomy (1), mastocytosis (1), asthma (2), cystic fibrosis (1), paralysis of the facial nerve (1), milk allergy (1). The complete list is shown in the Table 5.

**Table 5:** Reactions in children with underlying disease

**Symptoms for access**	**Vaccine causing the reaction**	**Underlying disease**
Urticaria	Unknown	Paralysis of the facial nerve
Lymphadenopathy	Unknown	Spherocytosis, splenectomy
Urticaria	DTPa	Mastocytosis
Local swelling	Hexavalent	Milk allergy
Convulsions	Hexavalent	Myoclonic epilepsy
Fainting	Hexavalent	Cystic fibrosis
Respiratory failure	Hexavalent	Prematurity, broncodisplasia
Thrombocytopenia	Hexavalent	Thrombocytopenia
Fever and urticaria	Hexavalent	UVR-renal hypoplasia
Fever and urticaria	hexavalent	UVR-renal hypoplasia
Fever	Hexavalent + pneumococcus	Connatal syphilis
Febrile convulsions	Hexavalent + pneumococcus	Asthma
Vomiting, dyspnea	Hexavalent + pneumococcus	Genetic disease-RGE
Urticaria	Hexavalent + pneumococcus	Atopy
Hypotonia	Hexavalent + pneumococcus	RGE
Fever	Hexavalent + pneumococcus	RGE
Convulsion 24 hours after	Hexavalent + pneumococcus	Tuberous sclerosis, West syndrome
Fever and urticaria	MMR	Asthma
Fever and urticaria	Pneumococcus	Thrombocytopenia
Uncontrollable crying	Pneumococcus	DiGeorge syndrome, VSD, RGE

**Figure 1:**
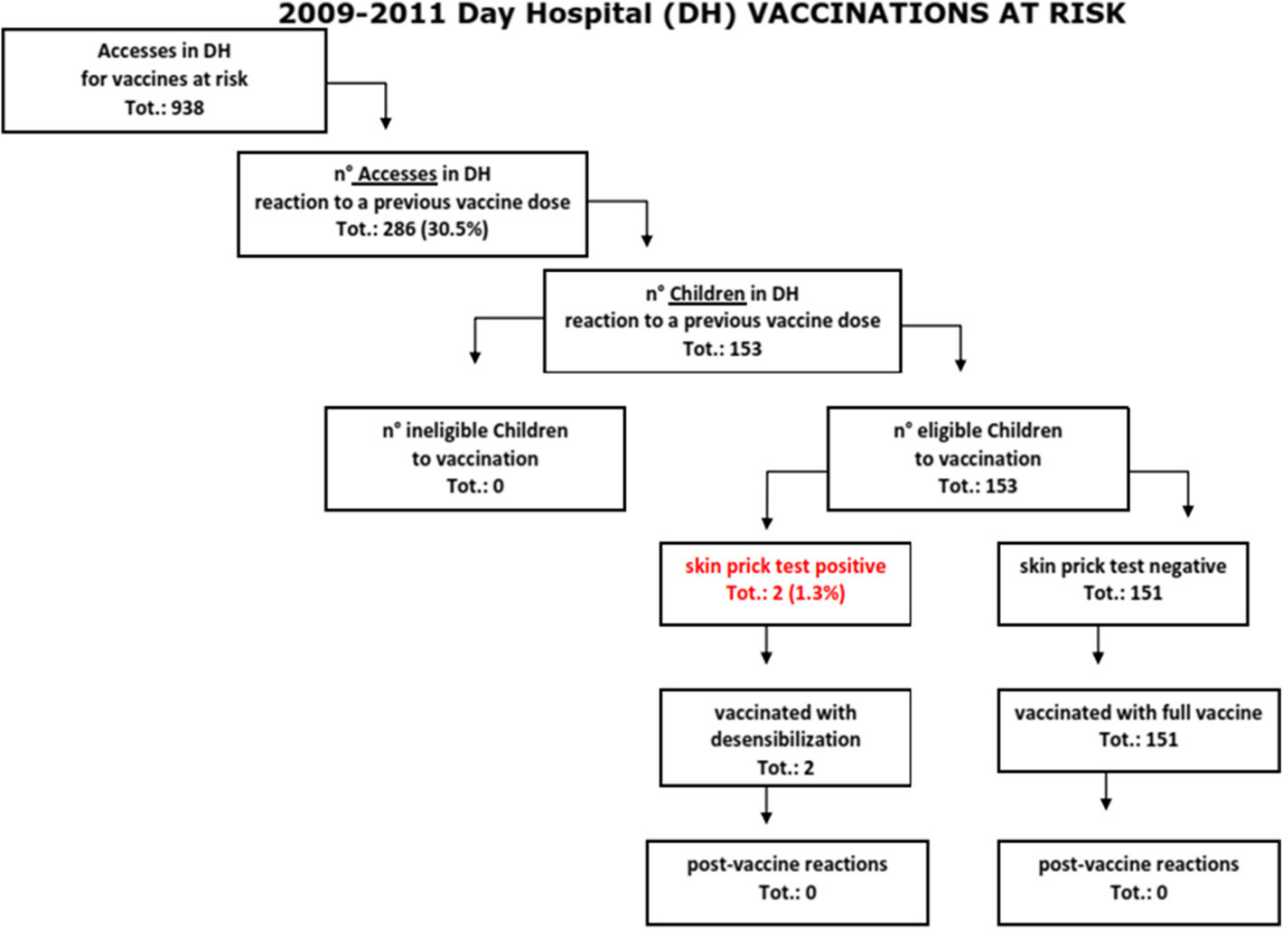
Algorithm for the management of an adverse reaction to vaccine.

Figure 1 shows the algorithm for the management of an adverse reaction to vaccine.

The hexavalent vaccine was the most reactogenic, almost always at the first dose, especially if co-administered with pneumococcus conjugate vaccine (73%). Numerically negligible reactions were caused by pneumococcus conjugate vaccine alone and MMR vaccine.

SPT were negative in 151 children (98.6%), despite the reaction to a previous vaccine dose; therefore full vaccine was administered; there were no reactions within 3 hours of observation.

Two cases had positive SPT; the first one was a child who presented 24 hours after first dose of hexavalent, fever, vomiting and hypotonia and had been redirected to the center by the Local Health Agencies to continue the vaccination schedule in a safe environment. This child came without other vaccines to our observation at the age of 7 months. After a communicating to his mother the procedures to undertake in the event of a positive SPT to hexavalent, she decided to delay the hexavalent, agreeing to practice the 1st dose of pneumococcal conjugate vaccine. Due to a referred allergic family history, we decided to make vaccination at hospital. As the SPT was positive (wheal 3 mm), we practiced desensitization inoculating the vaccine in escalating doses, 1/3 of the vial, the first two subcutaneously, the third intramuscular; after 3 hours of observation any reaction was registered. Then this child came back for the second dose of hexavalent. SPT was negative, so that the vaccine was administered in a single dose. The child did not manifest any reaction.

The second case presented IgE-mediate sympotms (urticaria and angioedema of the trunk and limbs) few minutes after 2nd dose of hexavalent + pneumococcal conjugate vaccine, which required access to the emergency room and treatment with cortisone.

SPT with hexavalent was practiced to the child for the third dose and resulted positive. After a discussion with the parents, it was decided not to perform the hexavalent but to schedule more accesses to safe environment for single vaccine separately. All individual SPT were negative, vaccines administered in full dose in a single solution, no reaction was recorded within 3 hours of observation.

Of the 153 children examined, whose parents reported symptoms perceived as severe reaction after vaccination, only 13 had symptoms suggestive of initial IgE-mediate sympotms with angioedema manifestations, only 2 of whom had positive screening a STP, which required alternative measures of administration of the vaccine. In none of the 2 cases post vaccination reaction was reported and the vaccination program was not stopped.

## Discussion

After an adverse event is recognized as a side effect of vaccination, assessing its seriousness is the next step. The profiles on the frequency and severity of side effects are established for each vaccine in the pre-release authorization phase and, above all, in the course of studies of post-release supervision. If deemed necessary, the serious side effects may include termination of licensure for the vaccine, especially if sufficient to cause an unfavorable risk-benefit [16].

A post-vaccination correct diagnosis is required for several reasons. First, if an adverse event is recognized as a contraindication to further doses of the same or similar vaccines, misdiagnosis can lead to unjustified cessation of an immunization series and this places the individual unnecessarily at increased risk for the vaccine-preventable disease. Second, without the use of solid, evidence-based and broadly accepted uniform case definitions, overdiagnosis or underdiagnosis of an event may be the result. This may affect the general perception of the safety profile of a given vaccine and, in the worst case, can endanger a whole immunization program. Over-diagnosis obviously can make a vaccine appear to be more dangerous than it really is while the opposite may happen with under-diagnosis. Third, comparability of safety profiles of vaccines in different studies or surveillance settings is extremely difficult if not impossible whether different case definitions for the same events are being used. This is especially problematic when rare events are being studied in meta-analyses [12].

Almost all recommended vaccinations either universally or for certain risk groups provide moderate to high levels of individual protection. The magnitude of protection can be expressed by the percent risk reduction to either develop a specific disease or a specific manifestation of a disease in comparison to an unimmunized individual [18]. This can either be performed during pre-licensure efficacy trials or during outbreaks. Without undisputable demonstration of its efficacy, a vaccine would neither be licensed nor recommended by responsible authorities.

If a vaccine is protecting from infection with a microorganism that is transmitted from human to human, not only direct protection of the individual can be expected but also indirect protection of unimmunised contact persons can be achieved if immunisation rates are high enough on a population level. This is the concept of the so called herd immunity that can be applied to the great majority of current standard immunisations with the notable and obvious exception of tetanus, which is not transmissible between humans [13].

All serious adverse events that occur after the administration of a vaccine should be reported to the competent authorities, even if it is not causal relationship between an adverse event and the vaccine.

The onset of clinical symptoms after vaccine administration not necessarily mean that the origin is always to be attributed to the vaccine. Most of the side effects of specific vaccinations turned out to be random observations. The differentiation between coincidence and causality is of utmost importance in this regard. The easiest way to prevent most adverse reactions is to identify during history taking pre-vaccination, the various precautions and contraindications.

The Italian National Institute of Health has published, last update in 2009, the “Guide to the contraindications to vaccination”, a full and important document easy to use [19]. The preparation of this fourth edition has been produced by a working group including more than 40 experts in immunization operating in major institutions in our Country (vaccination of local health services and the Regions, National Institute of Health, Ministry of Health, Cochrane Vaccines Field, universities, etc.). This guide is an adaptation and extension of that produced by the U.S. CDC.

The Guide is a reference tool for healthcare professionals working in the field of vaccinations and is intended to provide technical support for the proper assessment of contraindications or precautions to the vaccine administration. Some symptoms or conditions are mistakenly considered as true contraindications or situations that induce an attitude of precaution. These errors can lead to missed opportunities for vaccine administration, individual and social protection, by major diseases. Conversely, the administration of the vaccine in the presence of true contraindications or precautions may increase the risk of serious adverse reactions.

In our study, symptoms were reported by parents as they perceived them; an accurate history has allowed to classify them according to conventional canons. For this reason, we suggest that a specific and validated questionnaire should be prepared to collect data. In our experience, we found out a gap between true and reported adverse reactions. The over-diagnosis of adverse reactions to vaccines implies an inappropriate use of the hospital setting by pediatricians, by Local Health Agencies of Rome and Lazio and by children’s families.

In the cases of anaphylaxis history, a real danger has never been recorded, and this category was assigned to those cases of angioedemi with or without giant urticaria, rarely associated with respiratory manifestations type dyspnea, always mild. Seizures were always associated with post vaccination hyperpyrexia and have proved a posteriori isolated (simple febrile seizure). The overtone crisis and dyspnea were almost always associated with crying. The term “hyporesponsiveness” included what parents reported as “fainting” but in reality there has never been a loss of consciousness. The item “hives” also included those referred to as marbling and erythema spread to the whole body, not necessarily with appearance of papules on the skin. The risk/benefit ratio analysis in our patients has always been in favor of the net benefit of immunization, despite severe reactions have occurred in rare cases.

## Conclusions

Inadequate levels of immunization against infectious diseases remain a significant problem for public health. However, the reasons for incomplete vaccination and non-adoption of vaccination services are manifold.

To maintain public confidence in vaccines, advanced immunization programs must include activities for monitoring the safety of the vaccine at the individual level, then pursuing specialized counseling pre-and post-vaccination for those at risk [20,21], for a better specific risk/benefit ratio analysis, despite severe reactions may occur in rare cases.

A limit of our study is that patients were admitted to hospital for vaccination due to a self- reported diagnosis of a previous adverse vaccine reactions. Moreover, we collected anamnestic informations, without using a specific and validated questionnaire. The most important result is that we found out a gap between true and reported adverse reactions, which led to an inappropriate use of the hospital setting.

## Competing interests

The authors declare no founding, competing interest, and ethical approval.

## Authors’ contributions

LN and AC wrote the manuscript; AVittucci, EB, AG and RM contributed to collect data and carried out the tables; AVillani reviewed the manuscript. All authors read and approved the final manuscript.
